# Mediterranean Diet, Physical Activity and Gut Microbiome Composition: A Cross-Sectional Study among Healthy Young Italian Adults

**DOI:** 10.3390/nu12072164

**Published:** 2020-07-21

**Authors:** Francesca Gallè, Federica Valeriani, Maria Sofia Cattaruzza, Gianluca Gianfranceschi, Renato Liguori, Martina Antinozzi, Beatriz Mederer, Giorgio Liguori, Vincenzo Romano Spica

**Affiliations:** 1 Department of Movement Sciences and Wellbeing, University of Naples “Parthenope”, 80133 Naples, Italy; francesca.galle@uniparthenope.it (F.G.); giorgio.liguori@uniparthenope.it (G.L.); 2 Department of Movement, Human and Health Sciences, University of Rome “Foro Italico”, 00135 Rome, Italy; federica.valeriani@uniroma4.it (F.V.); gianluca.gianfranceschi@uniroma4.it (G.G.); 3 Department of Public Health and Infectious Diseases, Sapienza University, 00185 Rome, Italy; mariasofia.cattaruzza@uniroma1.it (M.S.C.); martina.antinozzi@uniroma1.it (M.A.); 4 Department of Sciences and Technologies, University of Naples “Parthenope”, 80143 Naples, Italy; renato.liguori@uniparthenope.it; 5 Department of Didactics of Language and Literatura, University of Granada, 18150 Granada, Spain; bmederer@correo.ugr.es

**Keywords:** gut microbiome, body mass index, Mediterranean diet, physical activity, *Firmicutes*, *Bacteroidetes*

## Abstract

Background. This cross-sectional study aimed to explore the microbial composition of the gut and its possible association with the Mediterranean diet (MD) after adjusting for demographic and anthropometric characteristics in a sample of healthy young Italian adults. Methods. Gut microbiota, demographic information, and data on adherence to MD and physical activity (PA) habits were collected in a sample of 140 university students (48.6% males, mean age 22.5 ± 2.9) with a mean body mass index (BMI) of 22.4 ± 2.8 kg/m^2^ (15.2–33.8) and a mean PA level of 3006.2 ± 2973.6 metabolic equivalent (MET)-minutes/week (148–21,090). Results. A high prevalence of *Firmicutes* and *Bacteroidetes* was found in all the fecal samples. Significant dissimilarities in the microbiota composition were found on the basis of MD adherence and PA levels (*p* = 0.001). At the genus level, *Streptococcus* and *Dorea* were highly abundant in overweight/obese individuals, *Ruminococcus* and *Oscillospira* in participants with lower adherence to MD, and *Lachnobacterium* in subjects with low levels of PA (*p* = 0.001). A significantly higher abundance of *Paraprevotella* was shown by individuals with lower BMI, lower MD adherence, and lower PA levels (*p* = 0.001). Conclusions. This study contributes to the characterization of the gut microbiome of healthy humans. The findings suggest the role of diet and PA in determining gut microbiota variability.

## 1. Background

Recent evidence has shown that gut microbiota, depending on its composition, may influence human health and diseases [1,2] and that it can be influenced by host and environmental factors [3,4,5]. In healthy adults, two bacterial phyla have been recognized as predominant: the Gram-positive *Firmicutes* and the Gram-negative *Bacteroidetes* [6]. However, the *Firmicutes*/*Bacteroidetes* (F/B) ratio has been observed to change with the nutritional status, age, and gender [3,5,7,8,9]. In particular, it was shown to be associated with the body mass index (BMI), since it tends to be higher in obese people and to decrease with weight loss [7,10,11].

Diet and physical activity (PA) are likely to play a role—in particular, diet is a fundamental factor in determining which nutrients gut microbes can use for their biological processes, and the metabolic products of those processes may have important impacts on health and disease [12]. In particular, the Mediterranean diet (MD), due to its inclusion of non-refined cereals, fruits and vegetables, olive oil, and red wine, may favor the presence of some bacteria in the gut, such as *Bifidobacterium* and *Lactobacobacillus*, which are typically health-related [13]. MD seems to be the best pattern to beneficially modulate gut microbiota biodiversity and, consequently, prevent several disorders, including gastrointestinal or even neurological disease, through the gut-brain axis [14,15,16]. The relationship between PA and the gut microbiota is still debated, but evidence shows that exercise may foster specific gut microbial profiles, especially among lean individuals [17,18].

A deeper understanding of the characteristics associated with gut microbiota composition in healthy people is necessary to improve knowledge about its role in physiological and pathological processes. Thus, the present study aimed to explore the bacterial composition of fecal samples and its possible association with dietary and PA habits in a sample of healthy young Italian adults. In particular, the BMI and adherence to MD patterns were used as indicators of diet, while weekly activities were used to assess the habitual levels of PA.

## 2. Materials and Methods

### 2.1. Setting and Participants

The study protocol was approved by the institutional review board of the University of Rome “Foro Italico” (number CAR 10/2018) and tested in a previous pilot study [19]. Students attending the University of Naples “Parthenope” and University of Rome “La Sapienza” were invited to participate to the study at the end of their lessons. During the invitation, a researcher explained the aim and the procedures of the study and guaranteed anonymity in the collection and treatment of personal information. Those who agreed to adhere were asked to sign a written informed consent in accordance with the standards of the Helsinki Declaration.

### 2.2. Questionnaires

Participants were asked to report through a questionnaire their age, gender, weight, height, and particular diet regimens (i.e., vegetarian or vegan), which did not imply exclusion. Intercurrent chronic diseases, pregnancy, food intolerances, concomitant infections (and the associated use of antibiotics and probiotics), or gastrointestinal surgical procedures in the 3 previous months were also investigated and represented exclusion criteria. The 9-items questionnaire developed by Martínez-González et al. and the short version of the International Physical Activity Questionnaire (IPAQ) were used to assess the dietary habits and the habitual PA levels of the students, respectively [20,21]. The first is a short screener employed to assess the adherence to cardioprotective MD, derived by the 136-item questionnaire validated in a sample of Spanish adults [22]. It allows to attribute a value of 1 to each reported dietary habit corresponding to the MD pattern, resulting in a maximum total score of 9, which accounts for the highest adherence level. The IPAQ total score is expressed in metabolic equivalent (MET)-minutes/week.

### 2.3. Analysis of Fecal Samples

A fecal swab (Copan Italia S.P.A., Brescia, Italy) was given to each participant, together with the instructions for the collection of a stool sample; participants were asked to deliver the swab on a planned day within two hours since collection. Samples were then stored at 4–8 °C in a refrigerated container and were taken within 24 h to the laboratory of the University of Rome “Foro Italico”, where they were processed with a previously validated protocol for DNA extraction from fecal traces [23]. Feces were weighed prior to the extraction, and DNA was purified and normalized; the libraries for next-generation sequencing (NGS) were prepared according to the 16S Metagenomic Sequencing Library Preparation Guide (part# 15044223 rev A; Illumina, San Diego, CA, USA). The PCR amplicons were obtained using primers containing overhang adapters: Ba27F 5′-TCGTCGGCAGCGTCAGATGTGTATAAGAGACAGAGAGTTTGATCCTGGCTCAG-3′ and Ba338R 5′-GTCTCGTGGGCTCGGAGATGTGTATAAGAGACAGTGCTGCCTCCCGTAGGAGT-3′ [24,25]. Tagged PCR products were generated using primer pairs with unique barcodes through two-step PCR. In this strategy, target primers containing overhang adapters were used in the first PCR reaction to amplify the target gene, and that product was then used in the second PCR using primers-containing barcodes. Each amplification reaction had a total volume of 25 μL, containing 12.5 μL of KAPA HiFi Hot Start Ready Mix (Roche, Pleasanton, CA, USA), 5 μL of each primer (1 μM), and 2 μL of template DNA. Reactions were carried out on a Techne^®^ TC-PLUS thermocycler (VWR International, LLC, Radnor, PA, USA). Following amplification, 5 μL of PCR product from each reaction was used for agarose gel (1%) electrophoresis to confirm amplification. The final concentration of cleaned DNA amplicon was determined using the Qubit PicoGreen dsDNA BR assay kit (Invitrogen, Grand Island, NY, USA) and validated on a Bioanalyzer DNA 1000 chip (Agilent, Santa Clara, CA, USA). Libraries were prepared using the MiSeq Reagent Kit Preparation Guide (Illumina, San Diego, CA, USA). Raw sequence data was processed using an in-house pipeline that was built on the Galaxy platform and incorporated various software tools to evaluate the quality of the raw sequence data (FASTA/Q Information tools, Mothur). All datasets were rigorously screened to remove low-quality reads (short reads >200 nt, zero-ambiguous sequences). Demultiplexing was performed to remove PhiX sequences and sort sequences; moreover, to minimize sequencing errors and ensure sequence quality, the reads were trimmed based on the sequence quality score using Btrim (an average quality score of 30 from the ends, and remove reads that are less any 200 bp after end-trimming) [26]. OTUs (operational taxonomic units) were clustered at a 97% similarity level, and final OTUs were generated based on the clustering results, and taxonomic annotations of individual OTUs were based on representative sequences using RDP’s 16S Classifier 2.5. Observed OTUs were defined as observed species. A level of 97% sequence identity is often chosen as representative of a species and 95% for a genus. The sequence reads were analyzed, also, in the cloud environment BaseSpace through the 16S Metagenomics app (version 1.0.1; Illumina^®^): the taxonomic database used was the Illumina-curated version (May 2013 release of the Greengenes Consortium Database) [27]. The raw sequencing data were submitted to NCBI Sequence Read Archive with the project accession number of PRJNA630035.

### 2.4. Statistical Analysis

A descriptive analysis was carried out on the collected information. Weight and height values were used to calculate the BMI. Age, BMI, adherence to MD, and levels of habitual PA were expressed as mean ± SD and ranges; numbers and percentages of participants for each gender and BMI category as defined by the World Health Organization standards [28] were also reported. Participants were also grouped by MD scores (lower or equal/higher than the median value obtained in the whole sample) and by PA levels (low, moderate, or high) [29].

Relative abundances of community members were determined with rarefied data and summarized at each taxonomic level. The proportion of the gut microbiome at each taxonomic rank, such as phylum, order, class, family, and genus, was determined using the RDP classifier and the Greengenes Database. The standard pipeline for 16S rDNA amplicon analysis revealed that the bacteria fell into 29 phylotypes (similarity level = 97%). Briefly, the relative abundance of the bacterial community was determined on 29 phylotypes (% abundance = (number of sequences for phylum or genera/total sequences for sample) × 100). The subsequent mathematical analyses excluded the 24 rarest phyla—that is, those with a relative abundance below 0.1 % in all the samples. A total of 23,388,649 sequences were generated from 140 samples. The number of sequences for each sample ranged from 42,072 to 437,525, leading to the identification of 630 OTUs defined at 97% identity. Alpha and beta diversity were calculated using EstimateS software at a level of 97% sequence similarity. Regarding alpha diversity, the Shannon index and equitability index at the species level were computed [30,31]. Principal coordinates analysis (PCoA) was performed using the METAGENassist platform [32] in order to investigate the dissimilarity between the groups. To assess the sequencing depth, alpha rarefaction plots were done using the software Mothur (version 1.31.1, www.mothur.org) and R (version 3.1.3, www.R-project.org)) with packages “ggplot2” and “vegan” (R Core team 2013). The R package was employed for comparative analyses of taxonomic and functional microbiota composition. Heatmaps and clustering analyses were based on the Pearson’s correlation as a measure of the distance and Ward’s method using the METAGENassist platform [32].

Student’s *t*-test was used to compare the bacterial diversity and relative abundance of the main bacterial phyla and genus (accounting for the 90% of fecal bacterial component) of samples from participants with different BMI (included in the underweight/normal weight and overweight/obese groups) and levels of MD adherence. ANOVA with a Bonferroni post hoc test was employed to evaluate differences among participants grouped by habitual PA levels and to compare lifestyle subgroups obtained by merging MD adherence and PA. The statistical significance of factors potentially contributing to compositional differences among microbiota samples was tested also with the PRIMER software (version 7) by using the nonparametric permutation Analysis Of Similarity (ANOSIM) function and the default setting [33]. In order to identify possible determinants of the microbiome composition, two linear regression analyses were performed by considering age, gender, BMI, MD adherence, and PA level as independent variables and the Shannon index or F/B ratio as the outcomes. A stepwise procedure was used in order to adjust for possible confounders. A value of *p* < 0.05 was considered statistically significant. Data were analyzed with IBM SPSS version 25 for Windows (SPSS, Chicago, IL, USA).

## 3. Results

**Figure 1:**
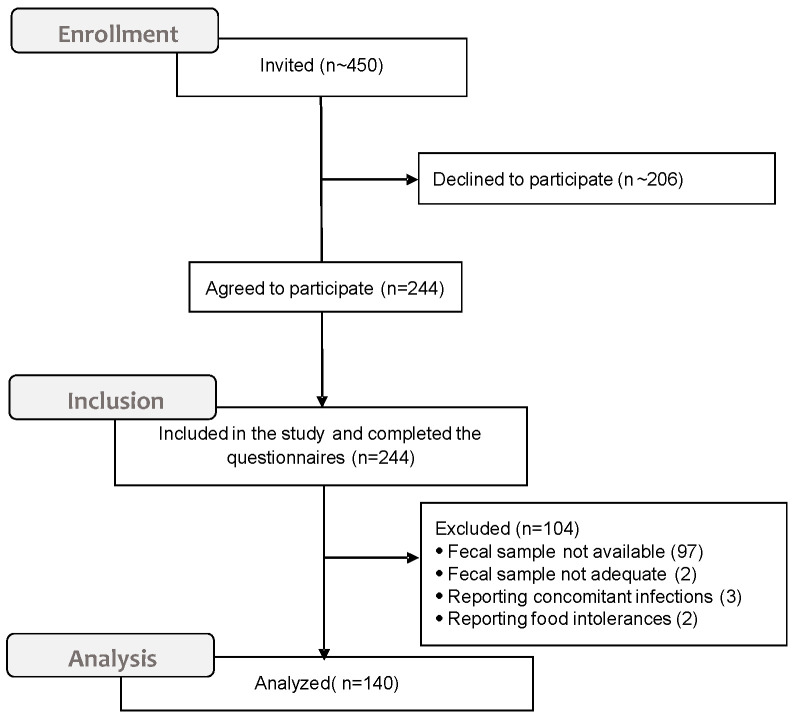
CONSORT diagram for the sample generation.

About 450 undergraduates attending classes were invited to take part in the study. Of a total of 244 (54.2%) students who initially agreed to participate, 140 (57.3%) completed the questionnaire, consigned the fecal swab on the planned date, and fulfilled the inclusion criteria (Figure 1).

Table 1 shows the main characteristics of the total sample. No particular diet regimens were reported by the participants.

**Figure 2:**
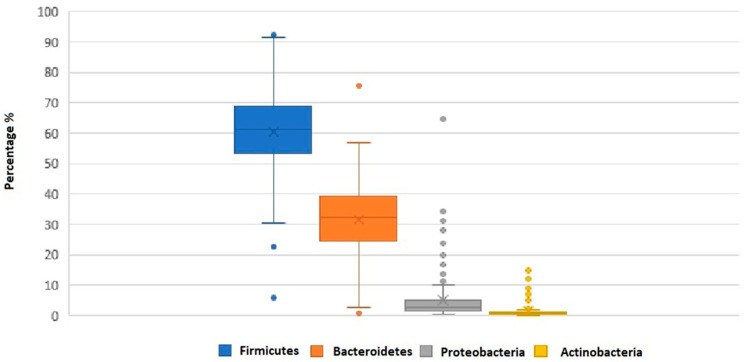
Box plot showing the relative abundance for the dominant phyla determined by the Global Alignment Sequence Taxonomy assignments. The boundary of the box closest to zero indicates the 25th percentile, the line within the box represents the median, and the boundary of the box farthest from zero indicates the 75th percentile. Whiskers above and below the box indicate the 10th and 90th percentiles. The arithmetic mean is indicated by x. Phyla less than 1% of the sequences were not reported.

Out of 28 bacterial phyla detected in the fecal samples, the highest abundances were registered for *Firmicutes* (61.6 ± 14.6) and *Bacteroidetes* (30.7 ± 13.3) (Figure 2).

Table 2 shows the differences in the Shannon index, *Firmicutes*, and *Bacteroidetes* relative abundances and F/B ratios between the BMI and MD score groups and among the PA groups, with corresponding *p*-values. No significant differences in variability nor in the phyla relative abundance were found.

In line with these results, also, the ANOSIM test yielded no significant dissimilarity for the BMI groups (*R* = −0.011, *p* = 0.5). In the regression analyses, only the male gender was significantly associated with the Shannon index (*p* = 0.02, odds ratio (OR) −0.08, confidence interval (CI) 95% −0.16–−0.01) and with the F/B ratio (*p* = 0.03, OR 0.50, CI 95% 0.08–0.93).

**Figure 3:**
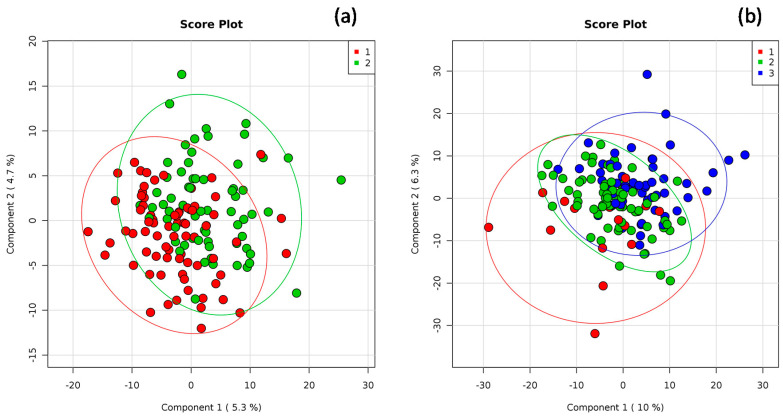
Principal coordinates analysis (PCoA) of the weighted Bray–Curtis for 16S rDNA data related to (**a**) the Mediterranean diet (MD) score: 1: low adherence (score ranging from 0 to 5) and 2: higher adherence to MD (score > 5) and (**b**) habitual physical activity (PA) level: 1: low, 2: moderate, and 3: high. Percentages on the axes represent the proportion of the variation explained by the 2 first components of the PCoA.

The global structure of the gut microbiota composition, as well as grouping patterns based on the MD score or PA level, are shown in Figure 3a and Figure 3b, respectively. As for the MD score, it is possible to observe slight differences also supported by the ANOSIM analysis (*R* value = 0.120, *p* = 0.001). The dissimilarity among the PA groups emerged from the principal component analysis, as shown in Figure 3b. The results of the ANOSIM indicated a slight dissimilarity among the three groups (*R* value = 0.350, *p* = 0.001).

Merging declared lifestyles, we obtained subgroups defined by the MD score and PA level. In the comparison of the abovementioned parameters among these lifestyle subgroups, no significant differences were detected (Table 3). The Bonferroni post hoc test also revealed no significant differences among the subgroups.

**Figure 4:**
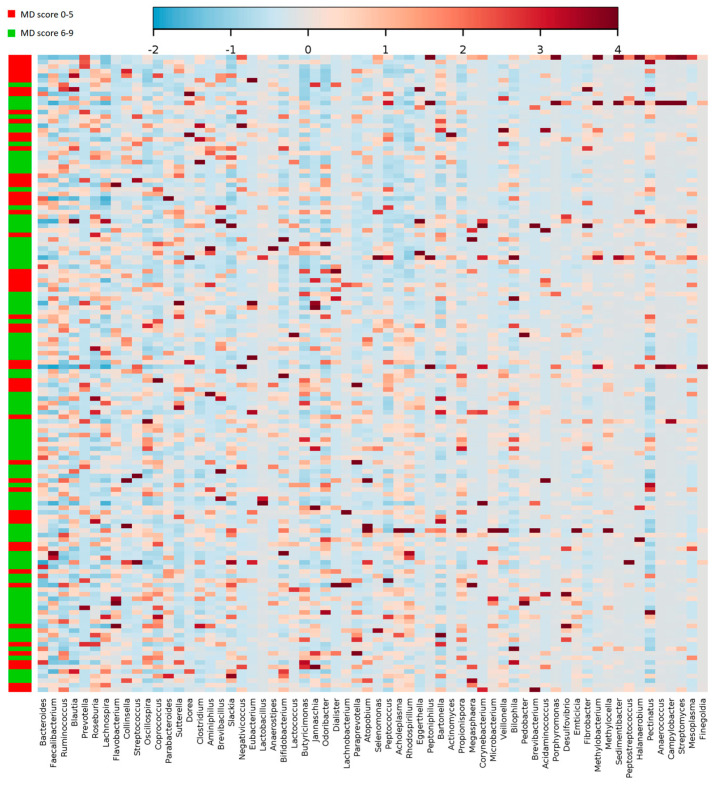
Heatmap and clustering of individual gut microbiota samples for taxonomic composition (genus level) in relation to the MD adherence. Clustering was based on Pearson distances and Ward’s linkage method. Only the genera above 2% abundance in at least one sample are depicted.

Plotting the correlation between the genera and adherence to the MD, an influence of the diet on the structure of the microbiota could be detected, although not strong in terms of absolute values (Figure 4). *Bacteroidetes* and *Prevotella* genera are the most represented in all fecal samples (19.8 ± 11.1 and 7 ± 13, respectively). However, no significant difference was detected between the MD score groups.

Table 4 shows the significant differences detected in the comparison of the genera relative abundances between the BMI and MD score categories and among the PA groups. The whole set of comparisons are reported in Supplementary Tables S1–S3. The *Streptococcus* and *Dorea* genera were highly represented in overweight/obese individuals, while *Ruminococcus* and *Oscillospira* were abundant among participants with lower adherence to MD. The *Lachnobacterium* genus was more abundant in subjects with low levels of PA. Furthermore, it is possible to observe some overlapping in these profiles. In particular, significantly higher amounts of *Megasphaera*, *Dialister*, and *Lachnobacterium* were found in overweight/obese and low-active subjects; a significantly higher abundance of *Paraprevotella* was shown also by individuals with lower BMI, lower MD adherence, and lower PA levels. The post-hoc Bonferroni test showed significant differences in genera abundance between the low PA group and the others.

## 4. Discussion

This study provides data on the composition of the gut microbiota in a Mediterranean population of young adults with different habits regarding MD and PA.

The results confirm the higher prevalence of *Firmicutes* and *Bacteroidetes* as prevalent phyla in the gut microbiota of healthy humans and further support the a role for diet and PA in determining the variability of the gut microbiota. Indeed, significant dissimilarities in fecal microbiota compositions were found between the MD groups and among the PA groups.

No significant differences in *Firmicutes* and *Bacteroidetes* abundances were observed on the basis of the BMI classification, as reported by Peters et al. [34]. As for the MD adherence, some studies performed on adults from Italy as well as other countries of the Mediterranean basin have shown the link between the MD and gut microbiota [13,35,36]. In line with the study by Mitsou et al. [13], we did not find significant differences related to diet in the relative amounts of the main phyla. However, our results were not in agreement with those from De Filippis et al. and Garcia-Mantrana et al., who reported an association between higher F/B ratios and a lower adherence to the MD or a high intake of animal-associated foods [35,36].

Nevertheless, at the genus level, several important differences were highlighted in our sample. In particular, as previously reported, the *Streptococcus* genus was highly abundant among overweight/obese participants, so as the *Dorea* and *Dialister*, which were also mainly represented among low-active individuals [37,38]. Interestingly, *Dorea* and *Dialister* were found to be related with insulin secretion and fasting blood glucose, suggesting their role in type 2 diabetes development in overweight-obese individuals [38]. Unlike what others have observed, *Oscillospira* was higher in subjects with a low adherence to MD patterns [37]. Lactic acid bacteria, such as the *Lactobacillus* and *Lactococcus* genera, were more represented in individuals who reported a higher adherence to the MD. This may be related to the dietary polyphenols, such as those present in olive oil, that represent substrates for Lactobacteria, mainly *Lactobacillus* strains, that are known to contributee in the healthy maintenance of the gut microbiota balance by modulating the oxidative status of the intestinal barrier, the inflammatory processes, and the immune response [39]. *Lachnospira* was more represented among individuals with higher adherence to the MD, while *Paraprevotella* was more represented in those with lower MD scores. The literature reports that vegetable-based diets tend to correlate with the amount of short-chain fatty acids (SCFA), which are produced by gut bacteria through dietary fiber fermentation and have fundamental effects on the immunity, metabolism, and inflammation processes of the host [38]. *Lachnospira* among *Firmicutes*, so as *Prevotella* among *Bacteroidetes*, seem to be involved in the fermentation of fibers, leading to a higher SCFA production. Even if the abundance of *Firmicutes* is generally associated with a higher intake of animal-associated foods, several members of this phylum were associated with SCFA production and were reported to be more abundant in individuals with higher adherence to the MD patterns and higher consumption of vegetables [17,39,40,41]. 

Our results did not show any correlation between *Prevotella* abundance and adherence to the MD pattern. Otherwise, the abundance of *Paraprevotella* genus was found to be significantly higher in the group with lower MD adherence, so as in less-active people, in accordance with previous studies [41,42]. Differences in the microbiota composition detected among the PA groups seem to confirm the role of exercise in determining the gut colonization by bacteria capable of SCFA production [17]. Furthermore, in our study, the relative abundance of *Ruminoccoccus* was found to be higher in subjects with a lower level of adherence to MD, and this is in line with the work of Martinez-Medina et al., showing an association between this genus and the consumption of a diet rich in saturated fat and sugars [43]. The *Megasphaera* genus was found to be more abundant in low-active subjects, in accordance to the findings from Liang et al., who reported a higher abundance of this genus in lower-level athletes in comparison with higher-level ones [40]. The higher abundance of *Lachnobacterium* in low-active participants is a novel observation that needs to be explored in depth. Interestingly, indeed, we observed several significant differences in genera abundance in the inactive group respect to the others, underlining a possible influence of physical activity in the presence of *Lachnobacterium* within the microbiota composition.

The analysis of biodiversity indexes and F/B ratio did not show any statistical significance among the different lifestyle subgroups. This could be due to the small sample size. Larger studies should be performed to verify these findings. In addition to the size of the sample, this study presents other limitations. First of all, the BMI, adherence to the MD, and the PA levels were calculated on the basis of self-reported information, and this could have led to inaccuracy or bias. Furthermore, we used a short and easy questionnaire to assess the MD adherence to facilitate the compliance of participants, but this did not allow us to perform a detailed analysis related to the quality and the quantity of nutrient intakes, e.g., fibers or animal proteins. However, our study offers an additional description of the microbiota composition in young adults from a Mediterranean population in association with their lifestyles and further studies are needed to characterize in depth the gut microbiota biodiversity in relation to several individual, social, and behavioral factors.

## 5. Conclusions

This study is a contribution to the characterization of the gut microbiome of healthy young Italian adults. It provides a picture of the relative abundance of the main bacterial phyla in the human gut and suggests the influence of diet and PA on the gut microbiota composition. From the perspectives of preventive medicine and public health, the results of this study underline the importance of promoting healthy behaviors in the population.

## Figures and Tables

**Table 1:** Main characteristics of the total samples.

	Participants
	*n* = 140
Age	
mean value ± SD (range)	22.5 ± 2.9 (18–36)
Gender	
*n (%)*	
males	68 (48.6)
females	72 (51.4)
BMI kg/m^2^	
mean value ± SD (range)	22.4 ± 2.8 (15.2–33.8)
BMI category	
*n (%)*	
under weight	7 (5.0)
normal weight	106 (75.7)
over weight	24 (17.1)
obese	3 (2.2)
MD adherence score	
mean value ± SD (median; range)	5.3 ± 1.6 (5; 2–9)
MD adherence level	
*n (%)*	
≤median	73 (52.1)
>median	67 (47.9)
Habitual PA	
MET-minutes/week	
mean value ± SD (range)	3006.2 ± 2973.6 (148–21,090)
PA level	
*n (%)*	
low	17 (12.1)
moderate	57 (40.7)
high	66 (47.1)

BMI, Body Mass Index, PA, physical activity, MD, Mediterranean diet, and MET, metabolic equivalent.

**Table 2:** Comparisons of the Shannon index, *Firmicutes*, *Bacteroidetes*, and the *Firmicutes*/*Bacteroidetes* (F/B) ratio of the BMI, MD score, and PA level groups, with corresponding *p*-values.

	**BMI**				***p*^a^**
	**Underweight/** **Normal Weight**		**Overweight**/ **Obese**		
Shannon Index	2.5 ± 0.2		2.5 ± 0.2		0.77
Firmicutes	58.9 ± 13.1		61.1 ± 8.7		0.47
Bacteroidetes	33.4 ± 10.4		31.8 ± 8.9		0.54
F/B ratio	2.1 ± 1.1		2.2 ± 1.3		0.56
	**MD score**				***p*^a^**
**Variable**	**0–5**		**6–9**		
Shannon Index	2.6 ± 0.2		2.6 ± 0.2		0.96
Firmicutes	58.9 ± 13.0		59.8 ± 11.9		0.68
Bacteroidetes	33.6 ± 11.1		32.6 ± 9.1		0.58
F/B ratio	2.1 ± 1.2		2.1 ± 1.0		0.94
	**PA level**				***p*^b^**
	**Low**	**moderate**		**High**	
Shannon Index	2.6 ± 0.1	2.6 ± 0.2		2.5 ± 0.2	0.46
Firmicutes	58.3 ± 16.0	58.1 ± 12.0		60.6 ± 11.8	0.55
Bacteroidetes	32.7 ± 14.8	34.6 ± 9.4		31.9 ± 9.4	0.38
F/B ratio	2.3 ± 1.5	1.9 ± 0.9		2.2 ± 1.1	0.27

^a^ Student’s *t*-test and ^b^ ANOVA.

**Table 3:** Comparisons of the Shannon Index, *Firmicutes*, *Bacteroidetes*, and F/B ratio of the subgroups categorized on the basis of MD adherence and PA levels, with corresponding *p*-values.

	Habitual PA Level	MD Adherence Score		*p*
		0–5 *n* = 73	6–9 *n* = 67	
Shannon Index	low active	2.6 ± 0.1	2.5 ± 0.2	
	moderately active	2.6 ± 0.2	2.6 ± 0.2	0.47
	highly active	2.5 ± 0.2	2.6 ± 0.2	
Firmicutes	low active	57.1 ± 17.1	63.8 ± 9.1	
	moderately active	59.0 ± 11.5	56.6 ± 12.9	0.51
	highly active	59.7 ± 12.6	61.1 ± 11.5	
Bacteroidetes	low active	32.8 ± 16.1	31.8 ± 8.4	
	moderately active	33.9 ± 9.1	35.6 ± 9.9	0.61
	highly active	33.4 ± 10.6	31.1 ± 8.6	
F/B ratio	low active	2.3 ± 1.6	2.1 ± 0.7	
	moderately active	1.9 ± 1.0	1.8 ± 0.9	0.81
	highly active	2.1 ± 1.3	2.1 ± 1.0	

**Table 4:** Relative abundance of significantly different genera in BMI, MD score, and PA groups.

**Genus**	**BMI**			***p*^a^**
	**Underweight/** **Normal Weight**		**Overweight/** **Obese**	
*Selenomonas*	0.23 ± 0.38		0.38 ± 1.30	0.02
*Megasphaera*	0.12 ± 0.29		0.32 ± 0.87	0.001
*Streptococcus*	1.66 ± 2.08		4.02 ± 6.69	0.001
*Dorea*	1.16 ± 2.29		2.44 ± 4.25	0.001
*Lachnobacterium*	0.27 ± 0.74		0.68 ± 2.43	0.007
*Jannaschia*	0.19 ± 0.31		0.27 ± 0.50	0.02
*Dialister*	0.35 ± 0.76		1.07 ± 2.34	0.001
*Eubacterium*	0.71 ± 2.19		1.61 ± 3.42	0.01
*Paraprevotella*	0.19 ± 0.30		0.08 ± 0.13	0.01
	**MD score**			***p*^a^**
**Genus**	**0–5**		**6–9**	
*Lachnospira*	0.37 ± 1.20		0.75 ± 2.28	0.02
*Oscillospira*	2.18 ± 1.90		1.73 ± 1.25	0.03
*Lactobacillus*	0.29 ± 0.52		1.26 ± 4.52	0.002
*Ruminococcus*	9.51 ± 6.69		7.63 ± 4.90	0.002
*Lactococcus*	0.22 ± 0.53		0.52 ± 1.30	0.01
*Veillonella*	0.09 ± 0.05		0.14 ± 0.17	0.001
*Paraprevotella*	0.23 ± 0.38		0.13 ± 0.22	0.008
	**PA level**			***p*^b^**
**Genus**	**low**	**moderate**	**high**	
*Megasphaera*	0.55 ± 1.08 ^1^	0.12 ± 0.24	0.11 ± 0.37	0.002
*Lachnobacterium*	1.30 ± 3.20 ^1^	0.25 ± 0.41	0.17 ± 0.25	0.001
*Dialister*	1.53 ± 2.69 ^1^	0.40 ± 0.80	0.24 ± 0.58	0.001
*Paraprevotella*	0.32 ± 0.49 ^2^	0.21 ± 0.29	0.12 ± 0.25	0.037

^a^ Student’s *t*-test and ^b^ ANOVA with Bonferroni post hoc test: ^1^ significantly different from moderate and high PA groups and ^2^ significantly different from the high PA group.

## Supplementary Materials

The following are available online at https://www.mdpi.com/2072-6643/12/7/2164/s1, Table S1: Mean abundance values ± SD of bacterial genera from fecal samples categorized per BMI category of participants. * *p* < 0.05, Table S2: Mean abundance values ± SD of bacterial genera from fecal samples categorized per MD score of participants. * *p* < 0.05, Table S3: Mean abundance values ± SD of bacterial genera from fecal samples categorized per PA level of participants. * *p* < 0.05.
