# Supplementary material for: Mediterranean Diet, Physical Activity and Gut Microbiome Composition: A Cross-Sectional Study among Healthy Young Italian Adults
Source: Nutrients. 2020 Jul 21;12(7):2164. doi: 10.3390/nu12072164 (PMC7401267; doi:10.3390/nu12072164)
Supplement: Supplementary file 1 [file nutrients-12-02164-s001.pdf]

Supplementary Table 1. Mean abundance values ± SD of bacterial genera from fecal samples categorized per BMI category of participants. * $p < 0.05$.

	BMI			
	Underweight/Normal weight		Overweight/Obese	
	Mean	Standard deviation	Mean	Standard deviation
Prevotella	4.62	8.22	3.93	8.05
Bacteroides	21.95	11.48	22.01	12.15
Sedimentibacter	.17	.95	.19	.68
Porphyromonas	.21	.97	.05	.10
Peptoniphilus	.38	1.19	.37	.92
Halanaerobium	.15	.55	.02	.05
Corynebacterium	.55	1.82	.63	1.83
Negativicoccus	.56	1.28	.44	.86
Faecalibacterium	12.57	6.75	13.30	7.56
Anaerococcus	.22	1.40	.15	.48
Oscillospira	2.05	1.69	1.59	1.39
Blautia	8.10	4.66	8.52	4.85
Flavobacterium	2.99	3.69	1.87	1.72
Roseburia	5.22	4.97	4.25	4.48
Campylobacter	.10	.50	.02	.05
Lactobacillus	.77	3.32	.72	2.59
Clostridium	1.28	1.10	1.21	.74
Ruminococcus	8.70	5.89	8.37	6.66
Lachnospira	4.03	2.09	4.93	2.56
Methylobacterium	.05	.15	.06	.11
Selenomonas	.23	.38	.38*	1.30
Aminiphilus	.92	1.95	.59	1.20
Megasphaera	.12	.30	.32*	.88
Coprococcus	1.33	1.53	1.02	1.11
Fusobacterium	.02	.08	.08<br>A	.20
Parabacteroides	2.17	3.27	1.83	1.92
Sutterella	.78	.89	.95	1.57
Streptomyces	.02	.07	.01	.02
Arcanobacterium	.01	.06	.00	.01
Streptococcus	1.66	2.09	4.03*	6.70
Actinomyces	.10	.15	.40	1.23
Peptostreptococcus	.02	.05	.12	.37
Mobiluncus	.02	.11	.01	.02
Collinsella	1.96	3.37	1.47	2.33
Acidaminococcus	.09	.28	.07	.16
Dorea	1.17	2.29	2.44*	4.26
Lachnobacterium	.28	.75	.68*	2.43
Finegoldia	.08	.66	.04	.11
Odoribacter	.23	.21	.21	.21
Borrelia	.00	.02	.00	.00
Brevibacterium	.10	.22	.05	.03
Peptococcus	.13	.12	.14	.10
Slackia	.69	.52	.82	.61
Facklamia	.03	.16	.00	.00
Propionispora	.12	.13	.10	.08

Bilophila	.05	.07	.08	.12
Atopobium	.24	.32	.20	.22
Brevibacillus	.73	1.20	.57	.72
Butyricimonas	.21	.18	.16	.18
Jannaschia	.19	.32	.28*	.50
Lactococcus	.34	1.01	.51	.95
Candidatus Phytoplasma	.09	.14	.06	.04
Dialister	.35	.77	1.07*	2.34
Megamonas	.30	2.53	.49	2.21
Veillonella	.11	.14	.11	.07
Ureaplasma	.00	.01	.00	.01
Rhodospirillum	.15	.12	.15	.11
Mesoplasma	.01	.01	.00	.01
Pectinatus	.02	.01	.02	.02
Fibrobacter	.03	.06	.02	.02
Tenacibaculum	.02	.05	.00	.00
Bartonella	.10	.10	.07	.09
Methylocella	.05	.15	.03	.01
Emticicia	.05	.07	.04	.02
Bifidobacterium	.26	.29	.29	.28
Sediminibacillus	.01	.07	.00	.00
Eggerthella	.12	.19	.14	.31
Acholeplasma	.14	.16	.12	.07
Microbacterium	.10	.23	.08	.05
Anaerostipes	.27	.66	.16	.17
Desulfovibrio	.07	.14	.03	.02
Pedobacter	.08	.16	.04	.05
Eubacterium	.71	2.20	1.61*	3.43
Mycobacterium	.01	.01	.01	.01
Paraprevotella	.19	.30	.08*	.13
Varibaculum	.04	.27	.01	.04
Paucibacter	.01	.01	.02*	.06
Mogibacterium	.04	.07	.04	.07
Anaerotruncus	.04	.05	.08*	.12
Chlorobaculum	.01	.01	.00	.00
Thermoanaerobacterium	.00	.01	.00	.00
Desulfosporosinus	.03	.07	.05	.07
Trabulsiella	.15	.45	.08	.21
Adlercreutzia	.15	.55	.07	.09
Brachybacterium	.00	.03	.00	.00
Paenibacillus	.03	.08	.01	.01
Pseudomonas	.60	3.48	.04	.13
Salinicoccus	.31	1.05	.32	.58
Turicibacter	.24	1.26	.12	.24
Enterococcus	.64	1.53	.44	.81
Lautropia	.02	.08	.01	.02
Stenoxybacter	.01	.02	.01	.01
Dysgonomonas	.01	.04	.01	.01
Geobacillus	.04	.05	.03	.02
Actinobaculum	.02	.14	.01	.02
Alkaliphilus	.04	.11	.05	.15
Caldicellulosiruptor	.00	.00	.01	.01
Fructobacillus	.05	.06	.04	.03
Natronincola	.04	.08	.03	.09

Phascolarctobacterium	.50	.81	.29	.49
Thermodesulfovibrio	.01	.02	.01	.01
Dethiosulfovibrio	.00	.01	.00	.00
Flammeovirga	.00	.00	.00	.00
Novosphingobium	.01	.03	.00	.00
Oxalobacter	.01	.02	.01	.03
Staphylococcus	.05	.23	.05	.17
Acetobacterium	.02	.11	.00	.01
Azospirillum	.02	.02	.02	.01
Sporosarcina	.05	.52	.00	.00
Syntrophomonas	.01	.02	.01	.01
Caldilinea	.01	.01	.01	.01
Coprobacillus	.29	1.05	.64	1.93
Jeotgalicoccus	.00	.03	.00	.00
Kitasatospora	.01	.02	.01	.01
Rhodanobacter	.00	.02	.01	.01
Zhouia	.05	.10	.03	.04
Carboxydocella	.00	.00	.00	.00
Carnobacterium	.03	.19	.01	.01
Chryseobacterium	.28	2.21	.01	.02
Enterobacter	.21	.66	.07	.13
Luteococcus	.00	.01	.00	.00
Marinobacter	.00	.01	.00	.00
Microvirus	.01	.01	.01	.01
Paracoccus	.02	.08	.01	.01
Roseospira	.02	.12	.01	.01
Sinorhizobium	.03	.12	.01	.02
Trichococcus	.06	.34	.02	.04
Amycolatopsis	.01	.05	.00	.00
Anaerofilum	.01	.02	.02	.04
Arcobacter	.07	.39	.00	.00
Brochothrix	.01	.01	.01	.01
Caulobacter	.01	.01	.00	.01
Luteibacter	.01	.03	.01	.01
Pseudoalteromonas	.01	.01	.01	.01
Thermicanus	.01	.01	.01	.01
Thiothrix	.00	.00	.00	.00
Achromobacter	.07	.45	.02	.07
Arthrobacter	.08	.78	.00	.00
Desulfonatronovibrio	.03	.05	.03	.02
Dyadobacter	.00	.02	.00	.00
Holdemania	.03	.05	.04	.06
Loktanella	.09	.33	.10	.47
Tetragenococcus	.01	.02	.01	.01
Weissella	.05	.21	.23	1.03
Brevundimonas	.12	.46	.06	.28
Candidatus Endobugula	.00	.01	.00	.00
Ectothiorhodospira	.00	.00	.00	.00
Exiguobacterium	.01	.01	.01	.01
Leptospira	.00	.03	.00	.00
Planifilum	.01	.03	.01	.01
Rickettsia	.00	.01	.00	.00
Sphingobium	.13	.61	.01	.02
Sphingomonas	.05	.36	.01	.01

Trichodesmium	.03	.11	.03	.06
Cystobacter	.00	.02	.00	.00
Deferribacter	.00	.00	.00	.00
Desulfotomaculum	.00	.00	.00	.00
Escherichia	.13	.37	.05	.09
Gemella	.02	.08	.18*	.80
Legionella	.00	.01	.00	.00
Pediococcus	.00	.00	.00	.00
Planomicrobium	.01	.02	.00	.00
Ralstonia	.01	.03	.00	.00
Rhodococcus	.04	.19	.00	.00
Thermobaculum	.00	.01	.00	.01
Thiomonas	.00	.01	.01	.02
Anaerofustis	.00	.00	.00	.00
Anaerovibrio	.00	.03	.00	.00
Asticcacaulis	.00	.01	.00	.01
Bacillus	.02	.12	.00	.00
Bradyrhizobium	.01	.03	.01	.02
Bulleidia	.01	.05	.02	.07
Citrobacter	.23	1.95	.02	.09
Cohnella	.02	.08	.02	.02
Coriobacterium	.00	.00	.01	.03
Devosia	.01	.06	.00	.00
Granulicatella	.01	.03	.02	.03
Haemophilus	.00	.01	.01	.02
Mitsuokella	.01	.10	.02	.11
Myroides	.01	.14	.00	.00
Niastella	.01	.02	.00	.00
Ochrobactrum	.00	.02	.00	.01
Parascardovia	.02	.03	.02	.02
Rhodobacter	.01	.03	.00	.00
Rikenella	.00	.00	.00	.00
Sarcina	.11	.35	.12	.27
Sphingopyxis	.01	.03	.00	.01
Succiniclasticum	.01	.08	.00	.00
Variovorax	.00	.01	.00	.00
Acidovorax	.09	.59	.00	.00
Acinetobacter	.08	.27	.04	.15
Aerococcus	.08	.88	.00	.00
Agrobacterium	.05	.27	.00	.00
Akkermansia	.01	.05	.02	.06
Alicycliphilus	.01	.07	.00	.00
Anoxybacillus	.07	.53	.00	.00
Burkholderia	.04	.17	.02	.05
Caloramator	.00	.00	.00	.00
Catenibacterium	.19	.76	.25	1.19
Chroococcus	.02	.06	.01	.01
Citricoccus	.00	.03	.00	.00
Cloacibacillus	.00	.04	.01	.05
Comamonas	.32	1.87	.00	.00
Curvibacter	.00	.01	.00	.00
Dechloromonas	.01	.04	.00	.00
Delftia	.22	1.11	.00	.00
Denitrobacter	.05	.44	.53	2.52

Erwinia	.00	.01	.01	.04
Ferrimicrobium	.00	.01	.00	.00
Fusibacter	.01	.04	.01	.03
Gallionella	.00	.01	.01	.02
Giesbergeria	.01	.04	.00	.00
Gluconacetobacter	.02	.16	.00	.00
Haloanella	.04	.39	.00	.00
Herbaspirillum	.00	.01	.00	.01
Hydrogenophaga	.02	.17	.00	.00
Klebsiella	.08	.68	.00	.01
Leptothrix	.00	.02	.00	.00
Leucobacter	.00	.01	.00	.01
Leuconostoc	.03	.22	.01	.03
Luteimonas	.01	.03	.00	.00
Macrococcus	.00	.01	.00	.01
Mesorhizobium	.01	.03	.01	.06
Methylibium	.02	.25	.00	.00
Mycoplana	.00	.03	.00	.00
Olivibacter	.01	.04	.00	.00
Phenylobacterium	.05	.55	.00	.00
Plesiomonas	.01	.06	.00	.00
Proteus	.01	.10	.00	.00
Providencia	.00	.01	.01	.01
Pseudochrobactrum	.01	.09	.00	.00
Pseudoclavibacter	.00	.02	.00	.00
Pseudoxanthomonas	.03	.27	.00	.00
Psychrobacter	.14	1.22	.00	.00
Rhizobium	.01	.02	.00	.01
Rhodoferax	.00	.03	.00	.00
Rhodoplanes	.01	.04	.00	.00
Rothia	.00	.01	.00	.01
Sediminibacterium	.00	.02	.00	.00
Serratia	.03	.16	.01	.01
Shinella	.01	.06	.00	.00
Sneathia	.00	.02	.19*	.73
Solibacillus	.04	.45	.00	.00
Sphingobacterium	.09	.44	.00	.00
Sporolactobacillus	.00	.01	.01	.00
Stenotrophomonas	.15	.77	.00	.01
Succinivibrio	.00	.04	.06*	.27
Thauera	.02	.17	.00	.00
Thermomonas	.03	.27	.00	.00
Vagococcus	.01	.04	.03	.15
Vibrio	.00	.01	.00	.00
Viridibacillus	.02	.10	.01	.05
Xanthomonas	.03	.21	.00	.00
Zobellia	.00	.00	.00	.01

Supplementary Table 2. Mean abundance values ± SD of bacterial genera from fecal samples categorized per MD score of participants. * $p < 0.05$.

	MD score			
	0-5		6-9	

	Mean	Standard deviation	Mean	Standard deviation
Prevotella	4.98	8.80	4.23	7.79
Bacteroides	22.39	11.62	21.33	11.41
Sedimentibacter	.14	.71	.20	1.07
Porphyromonas	.17	.72	.20	1.03
Peptoniphilus	.38	1.20	.38	1.08
Halanaerobium	.12	.52	.14	.48
Corynebacterium	.37	1.20	.75	2.28
Negativicoccus	.55	1.16	.58	1.31
Faecalibacterium	11.94	6.43	13.57	7.34
Anaerococcus	.27	1.64	.15	.73
Oscillospira	2.18	1.90	1.73*	1.26
Blautia	8.38	5.15	7.95	4.05
Flavobacterium	2.68	3.65	2.87	3.23
Roseburia	5.09	5.34	5.02	4.32
Campylobacter	.08	.35	.08	.54
Lactobacillus	.29	.52	1.26	4.53
Clostridium	1.18	.78	1.36	1.26
Ruminococcus	9.51	6.70	7.64	4.91
Lachnospira	0.37	0.20	0.75*	0.23
Methylobacterium	.05	.16	.05	.12
Selenomonas	.25	.43	.26	.79
Aminiphilus	.92	2.06	.81	1.59
Megasphaera	.20	.57	.13	.40
Coprococcus	1.36	1.48	1.16	1.45
Fusobacterium	.03	.10	.03	.12
Parabacteroides	2.33	3.99	1.86	1.52
Sutterella	.84	.96	.81	1.11
Streptomyces	.02	.06	.02	.06
Arcanobacterium	.02	.08	.00	.03
Streptococcus	1.99	3.64	2.09	3.11
Actinomyces	.18	.71	.11	.15
Peptostreptococcus	.03	.05	.05	.22
Mobiluncus	.02	.06	.02	.12
Collinsella	1.89	2.96	1.84	3.46
Acidaminococcus	.10	.29	.06	.21
Dorea	1.63	3.23	1.09	2.01
Lachnobacterium	.45	1.60	.22	.40
Finegoldia	.11	.83	.03	.12
Odoribacter	.24	.21	.21	.21
Borrelia	.00	.03	.00	.02
Brevibacterium	.08	.20	.10	.21
Peptococcus	.12	.09	.14	.14
Slackia	.71	.49	.70	.58
Facklamia	.02	.15	.02	.15
Propionispora	.12	.09	.11	.14
Bilophila	.06	.08	.05	.08
Atopobium	.23	.27	.23	.33
Brevibacillus	.65	.90	.74	1.34
Butyricimonas	.22	.19	.19	.17
Jannaschia	.18	.31	.23	.39
Lactococcus	.23	.53	.52*	1.31
Candidatus Phytoplasma	.07	.05	.11*	.17

Dialister	.55	1.46	.37	.81
Megamonas	.48	3.17	.17	1.29
Veillonella	.09	.05	.14*	.18
Ureaplasma	.00	.01	.00	.00
Rhodospirillum	.14	.08	.16	.14
Mesoplasma	.01	.01	.01	.02
Pectinatus	.02	.02	.02	.01
Fibrobacter	.03	.06	.03	.04
Tenacibaculum	.02	.06	.01	.02
Bartonella	.09	.10	.10	.10
Methylocella	.04	.04	.06	.19
Emticicia	.05	.02	.06	.09
Bifidobacterium	.26	.23	.29	.37
Sediminibacillus	.00	.02	.01	.09
Eggerthella	.12	.22	.13	.21
Acholeplasma	.13	.08	.13	.20
Microbacterium	.08	.07	.11	.29
Anaerostipes	.21	.25	.31	.84
Desulfovibrio	.07	.16	.05	.09
Pedobacter	.05	.05	.08	.21
Eubacterium	.71	2.19	1.04	2.69
Mycobacterium	.00	.01	.01	.01
Paraprevotella	.23	.38	.13*	.22
Varibaculum	.05	.32	.02	.12
Paucibacter	.01	.02	.01	.04
Mogibacterium	.04	.06	.04	.07
Anaerotruncus	.05	.08	.03	.05
Chlorobaculum	.01	.01	.01	.01
Thermoanaerobacterium	.00	.01	.00	.01
Desulfosporosinus	.03	.03	.04	.10
Trabulsiella	.14	.42	.13	.42
Adlercreutzia	.15	.62	.13	.34
Brachybacterium	.00	.01	.01	.04
Paenibacillus	.03	.10	.02	.02
Pseudomonas	.25	1.02	.84	4.45
Salinicoccus	.38	1.30	.23	.39
Turicibacter	.07	.17	.38	1.64
Enterococcus	.60	1.43	.64	1.46
Lautropia	.02	.05	.02	.10
Stenoxybacter	.01	.01	.01	.03
Dysgonomonas	.01	.01	.01	.05
Geobacillus	.03	.04	.04	.05
Actinobaculum	.02	.17	.00	.01
Alkaliphilus	.04	.09	.05	.15
Caldicellulosiruptor	.00	.00	.01	.01
Fructobacillus	.04	.04	.05	.07
Natronincola	.04	.10	.03	.06
Phascolarctobacterium	.39	.62	.53	.89
Thermodesulfovibrio	.01	.01	.01*	.03
Dethiosulfovibrio	.00	.01	.00	.01
Flammeovirga	.00	.00	.00	.00
Novosphingobium	.00	.01	.01	.03
Oxalobacter	.01	.02	.00	.01
Staphylococcus	.05	.21	.06	.22

Acetobacterium	.02	.13	.01	.06
Azospirillum	.02	.01	.02	.03
Sporosarcina	.08	.66	.01	.01
Syntrophomonas	.01	.01	.01	.02
Caldilinea	.01	.01	.01	.02
Coprobacillus	.54	1.63	.14	.45
Jeotgalicoccus	.00	.00	.01	.04
Kitasatospora	.01	.01	.01	.03
Rhodanobacter	.01	.03	.00	.01
Zhouia	.04	.06	.04	.12
Carboxydocella	.00	.00	.00	.00
Carnobacterium	.03	.23	.02	.05
Chryseobacterium	.32	2.67	.14	.81
Enterobacter	.19	.53	.19	.68
Luteococcus	.00	.00	.00	.01
Marinobacter	.00	.00	.01	.01
Microvirus	.01	.01	.01	.01
Paracoccus	.01	.01	.02	.10
Roseospira	.01	.01	.03	.16
Sinorhizobium	.03	.14	.02	.05
Trichococcus	.03	.08	.07	.44
Amycolatopsis	.01	.00	.01	.06
Anaerofilum	.01	.02	.01	.03
Arcobacter	.04	.28	.08	.43
Brochothrix	.01	.01	.01	.02
Caulobacter	.01	.01	.01	.01
Luteibacter	.01	.04	.01	.01
Pseudoalteromonas	.01	.01	.01	.02
Thermicanus	.01	.01	.01	.01
Thiothrix	.00	.00	.00	.00
Achromobacter	.04	.20	.09	.56
Arthrobacter	.11	.97	.01	.07
Desulfonatronovibrio	.03	.02	.03	.06
Dyadobacter	.00	.00	.01	.03
Holdemania	.03	.06	.03	.03
Loktanella	.11	.44	.06	.23
Tetragenococcus	.01	.02	.01	.01
Weissella	.10	.59	.05	.25
Brevundimonas	.05	.24	.17	.57
Candidatus Endobugula	.00	.00	.01	.01
Ectothiorhodospira	.00	.00	.00	.00
Exiguobacterium	.01	.01	.01	.01
Leptospira	.00	.00	.01	.04
Planifilum	.01	.02	.01	.03
Rickettsia	.00	.00	.00	.01
Sphingobium	.05	.31	.17	.73
Sphingomonas	.02	.03	.07	.47
Trichodesmium	.02	.09	.04	.12
Cystobacter	.00	.00	.00	.02
Deferribacter	.00	.00	.00	.00
Desulfotomaculum	.00	.00	.00	.00
Escherichia	.15	.44	.08	.17
Gemella	.03	.10	.06	.47
Legionella	.00	.00	.00	.01

Pediococcus	.00	.00	.00	.00
Planomicrobium	.01	.03	.00	.01
Ralstonia	.00	.00	.01	.04
Rhodococcus	.04	.16	.02	.18
Thermobaculum	.00	.01	.01	.01
Thiomonas	.00	.01	.00	.01
Anaerofustis	.00	.00	.00	.00
Anaerovibrio	.00	.00	.01	.04
Asticcacaulis	.00	.01	.01	.01
Bacillus	.03	.15	.00	.01
Bradyrhizobium	.01	.02	.01	.03
Bulleidia	.01	.06	.01	.04
Citrobacter	.01	.07	.39	2.56
Cohnella	.02	.04	.03	.10
Coriobacterium	.00	.02	.00	.00
Devosia	.00	.00	.01	.07
Granulicatella	.01	.02	.02	.04
Haemophilus	.00	.01	.00	.01
Mitsuokella	.00	.00	.03	.14
Myroides	.00	.00	.02	.18
Niastella	.00	.00	.01	.03
Ochrobactrum	.00	.02	.00	.02
Parascardovia	.01	.02	.02	.03
Rhodobacter	.01	.01	.02	.04
Rikenella	.00	.00	.00	.01
Sarcina	.07	.18	.15	.45
Sphingopyxis	.00	.02	.01	.04
Succiniclasticum	.01	.09	.01	.03
Variovorax	.00	.01	.00	.01
Acidovorax	.04	.18	.11	.75
Acinetobacter	.08	.24	.11	.42
Aerococcus	.00	.01	.14	1.15
Agrobacterium	.01	.03	.07	.35
Akkermansia	.01	.03	.01	.06
Alicycliphilus	.00	.02	.01	.09
Anoxybacillus	.11	.67	.00	.00
Burkholderia	.05	.21	.02	.05
Caloramator	.00	.00	.00	.00
Catenibacterium	.17	.79	.23	.89
Chroococcus	.02	.06	.01	.05
Citricoccus	.01	.04	.00	.00
Cloacibacillus	.01	.03	.01	.04
Comamonas	.13	.65	.41	2.36
Curvibacter	.00	.01	.00	.01
Dechloromonas	.00	.00	.01	.06
Delftia	.21	1.07	.15	.94
Denitrobacter	.23	1.50	.02	.16
Erwinia	.00	.00	.01	.03
Ferrimicrobium	.00	.00	.00	.01
Fusibacter	.01	.01	.01	.05
Gallionella	.01	.01	.01	.01
Giesbergeria	.01	.05	.00	.01
Gluconacetobacter	.00	.00	.03	.21
Haloanella	.00	.00	.06	.51

Herbaspirillum	.00	.01	.01	.01
Hydrogenophaga	.00	.00	.03	.22
Klebsiella	.12	.86	.01	.03
Leptothrix	.00	.00	.00	.02
Leucobacter	.00	.01	.00	.01
Leuconostoc	.02	.06	.05	.28
Luteimonas	.00	.01	.01	.03
Macrococcus	.01	.02	.00	.00
Mesorhizobium	.01	.04	.00	.03
Methylibium	.00	.00	.04	.33
Mycoplana	.00	.01	.00	.04
Olivibacter	.00	.03	.01	.05
Phenylobacterium	.08	.69	.00	.03
Plesiomonas	.01	.06	.01	.05
Proteus	.00	.00	.02	.13
Providencia	.00	.00	.00	.01
Pseudochrobactrum	.01	.11	.00	.01
Pseudoclavibacter	.00	.01	.00	.02
Pseudoxanthomonas	.01	.04	.05	.35
Psychrobacter	.00	.00	.24	1.60
Rhizobium	.01	.02	.01	.03
Rhodoferax	.00	.00	.01	.04
Rhodoplanes	.00	.01	.01	.05
Rothia	.00	.00	.00	.01
Sediminibacterium	.00	.00	.00	.02
Serratia	.02	.03	.04	.21
Shinella	.01	.04	.01	.07
Sneathia	.06	.41	.00	.03
Solibacillus	.07	.56	.00	.00
Sphingobacterium	.03	.23	.12	.53
Sporolactobacillus	.01	.01	.00	.00
Stenotrophomonas	.11	.62	.16	.78
Succinivibrio	.02	.15	.01	.06
Thauera	.00	.00	.03	.22
Thermomonas	.00	.00	.04	.35
Vagococcus	.00	.01	.02	.10
Vibrio	.00	.01	.00	.01
Viridibacillus	.03	.13	.01	.04
Xanthomonas	.01	.05	.04	.28
Zobellia	.00	.01	.00	.00

Supplementary Table 3. Mean abundance values ± SD of bacterial genera from fecal samples categorized per PA level of participants. * $p < 0.05$.

	PA level					
	low		moderate		high	
	Mean	Standard deviation	Mean	Standard deviation	Mean	Standard deviation
Prevotella	3.68	5.38	4.96	9.26	4.57	8.15
Bacteroides	22.86	12.42	22.17	12.37	21.38	10.58
Sedimentibacter	.04	.08	.14	.79	.23	1.08
Porphyromonas	.07	.20	.21	.86	.19	1.00
Peptoniphilus	.15	.08	.44	1.35	.38	1.09
Halanaerobium	.03	.05	.21	.70	.08	.30

Corynebacterium	.52	1.60	.65	1.79	.47	1.88
Negativicoccus	.43	.92	.44	1.14	.71	1.37
Faecalibacterium	13.44	8.62	12.53	6.38	12.70	6.96
Anaerococcus	.03	.05	.33	1.86	.16	.73
Oscillospira	2.10	1.59	2.24	2.03	1.69	1.19
Blautia	7.31	4.89	8.29	5.11	8.30	4.19
Flavobacterium	2.36	1.95	3.19	3.95	2.52	3.27
Roseburia	4.44	3.44	4.38	4.02	5.81	5.72
Campylobacter	.01	.01	.11	.39	.09	.55
Lactobacillus	.11	.12	.79	4.27	.88	2.40
Clostridium	1.01	.63	1.16	.75	1.42	1.29
Ruminococcus	8.97	6.99	8.27	5.75	8.83	5.94
Lachnospira	4.82	2.60	4.16	2.38	4.11	1.91
Methylobacterium	.02	.03	.05	.18	.06	.12
Selenomonas	.13	.22	.20	.39	.34	.83
Aminiphilus	1.14	1.89	.77	2.05	.88	1.65
Megasphaera	.55*	1.08	.12	.24	.11	.37
Coprococcus	.96	1.14	1.55	1.73	1.09	1.25
Fusobacterium	.03	.09	.04	.11	.03	.12
Parabacteroides	3.62	7.36	2.00	2.12	1.81	1.49
Sutterella	.71	.90	.79	1.13	.88	.98
Streptomyces	.01	.01	.02	.07	.02	.06
Arcanobacterium	.01	.02	.02	.09	.00	.01
Streptococcus	2.44	6.05	1.99	3.24	1.98	2.53
Actinomyces	.07	.08	.11	.17	.20	.74
Peptostreptococcus	.03	.04	.05	.24	.03	.06
Mobiluncus	.00	.01	.02	.08	.02	.12
Collinsella	2.47	2.85	1.56	2.45	1.97	3.81
Acidaminococcus	.13	.22	.05	.15	.10	.33
Dorea	.72	.57	.97	1.39	1.88	3.68
Lachnobacterium	1.31*	3.21	.25	.41	.17	.26
Finegoldia	.01	.02	.14	.94	.02	.10
Odoribacter	.22	.19	.24	.20	.22	.22
Borrelia	.00	.00	.01	.03	.00	.02
Brevibacterium	.15	.42	.08	.07	.09	.21
Peptococcus	.09	.07	.13	.12	.13	.12
Slackia	.77	.55	.75	.56	.65	.51
Facklamia	.00	.00	.05	.23	.01	.03
Propionispora	.11	.10	.12	.10	.11	.14
Bilophila	.10	.12	.05	.06	.05	.08
Atopobium	.27	.26	.20	.20	.26	.38
Brevibacillus	.61	.78	.52	.68	.86	1.45
Butyricimonas	.28	.27	.21	.18	.17	.14
Jannaschia	.27	.50	.25	.40	.15	.25
Lactococcus	.09	.13	.38	1.31	.42	.77
Candidatus Phytoplasma	.05	.04	.09	.10	.10	.16
Dialister	1.54*	2.70	.40	.80	.24	.58
Megamonas	.25	.85	.49	3.55	.21	1.33
Veillonella	.09	.04	.10	.07	.13	.17
Ureaplasma	.00	.00	.00	.01	.00	.01
Rhodospirillum	.16	.10	.16	.08	.14	.14
Mesoplasma	.00	.01	.01	.01	.01	.02
Pectinatus	.01	.01	.02	.01	.02	.02
Fibrobacter	.03	.04	.02	.03	.04	.07

Tenacibaculum	.00	.02	.02	.06	.01	.03
Bartonella	.08	.08	.08	.10	.11	.11
Methylocella	.05	.04	.04	.04	.06	.19
Emticicia	.04	.01	.05	.02	.05	.09
Bifidobacterium	.27	.29	.27	.30	.28	.31
Sediminibacillus	.00	.00	.01	.04	.01	.09
Eggerthella	.10	.12	.09	.11	.16	.28
Acholeplasma	.12	.06	.15	.09	.12	.19
Microbacterium	.06	.04	.10	.11	.10	.28
Anaerostipes	.23	.20	.18	.21	.33	.86
Desulfovibrio	.02	.03	.09	.18	.04	.08
Pedobacter	.06	.05	.10	.23	.04	.05
Eubacterium	.54	1.36	1.18	2.88	.69	2.23
Mycobacterium	.00	.01	.01	.01	.00	.01
Paraprevotella	.33*	.49	.21	.30	.12	.26
Varibaculum	.00	.01	.05	.35	.03	.14
Paucibacter	.01	.01	.01	.04	.01	.01
Mogibacterium	.03	.05	.05	.07	.04	.07
Anaerotruncus	.06	.11	.04	.07	.04	.05
Chlorobaculum	.00	.01	.01	.01	.01	.01
Thermoanaerobacterium	.00	.00	.00	.01	.00	.01
Desulfosporosinus	.03	.02	.04	.05	.03	.09
Trabulsiella	.29	.53	.11	.47	.12	.33
Adlercreutzia	.38	1.27	.06	.08	.14	.35
Brachybacterium	.00	.00	.00	.01	.01	.04
Paenibacillus	.02	.02	.04	.11	.02	.02
Pseudomonas	.03	.10	.29	1.16	.86	4.48
Salinicoccus	.63	2.36	.22	.44	.30	.68
Turicibacter	.06	.12	.10	.21	.37	1.65
Enterococcus	.34	.47	.84	1.79	.50	1.25
Lautropia	.01	.02	.02	.05	.02	.10
Stenoxybacter	.01	.00	.01	.01	.01	.03
Dysgonomonas	.01	.01	.02	.06	.01	.01
Geobacillus	.03	.03	.02	.02	.05*	.06
Actinobaculum	.00	.00	.00	.02	.03	.18
Alkaliphilus	.07	.17	.02	.02	.05	.15
Caldicellulosiruptor	.00	.00	.00	.00	.01	.01
Fructobacillus	.04	.03	.04	.03	.05	.08
Natronincola	.02	.01	.04	.11	.03	.06
Phascolarctobacterium	.24	.42	.44	.83	.53	.76
Thermodesulfovibrio	.00	.00	.01	.02	.01	.02
Dethiosulfovibrio	.00	.00	.00	.02	.00	.00
Flammeovirga	.00	.01	.00	.00	.00	.00
Novosphingobium	.00	.00	.00	.01	.01	.03
Oxalobacter	.01	.01	.01	.02	.01	.02
Staphylococcus	.11	.41	.06	.24	.03	.10
Acetobacterium	.00	.00	.03	.16	.01	.03
Azospirillum	.02	.01	.02	.01	.02	.03
Sporosarcina	.00	.01	.10	.74	.01	.01
Syntrophomonas	.01	.01	.01	.03	.01	.01
Caldilinea	.01	.01	.02*	.02	.01	.01
Coprobacillus	.12	.30	.30	1.15	.44	1.44
Jeotgalicoccus	.00	.00	.01	.05	.00	.00
Kitasatospora	.01	.01	.01	.01	.01	.03

Rhodanobacter	.01	.05	.00	.01	.01	.01
Zhouia	.06	.07	.04	.06	.04	.12
Carboxydocella	.00	.00	.00	.00	.00	.00
Carnobacterium	.00	.00	.05	.26	.01	.05
Chryseobacterium	.00	.00	.56	3.13	.01	.02
Enterobacter	.25	.49	.13	.45	.23	.73
Luteococcus	.00	.00	.00	.00	.00	.01
Marinobacter	.00	.00	.00	.01	.01	.01
Microvirus	.02	.01	.02*	.01	.01	.01
Paracoccus	.01	.01	.02	.11	.01	.03
Roseospira	.01	.00	.02	.02	.03	.16
Sinorhizobium	.01	.02	.03	.16	.02	.05
Trichococcus	.03	.08	.02	.02	.08	.45
Amycolatopsis	.01	.01	.01	.00	.01	.06
Anaerofilum	.01	.02	.01	.02	.01	.03
Arcobacter	.00	.00	.09	.47	.04	.29
Brochothrix	.01	.00	.01	.02	.01	.01
Caulobacter	.00	.00	.00	.01	.01	.02
Luteibacter	.02	.07	.00	.01	.01	.01
Pseudoalteromonas	.01	.01	.01	.02	.01	.01
Thermicanus	.00	.00	.01	.01	.01	.01
Thiothrix	.00	.00	.00	.00	.00	.00
Achromobacter	.03	.08	.05	.23	.08	.56
Arthrobacter	.00	.00	.16	1.10	.00	.02
Desulfonatronovibrio	.02	.02	.03	.02	.03	.07
Dyadobacter	.00	.00	.01	.03	.00	.00
Holdemania	.04	.04	.03	.07	.03	.03
Loktanella	.04	.16	.06	.28	.13	.44
Tetragenococcus	.01	.00	.01	.01	.01	.02
Weissella	.04	.16	.02	.09	.14	.65
Brevundimonas	.11	.43	.05	.16	.16	.57
Candidatus Endobugula	.00	.00	.00	.01	.00	.00
Ectothiorhodospira	.00	.00	.00	.00	.00	.00
Exiguobacterium	.01	.01	.01	.01	.01	.01
Leptospira	.00	.00	.01	.03	.00	.02
Planifilum	.01	.01	.01	.01	.01	.03
Rickettsia	.00	.01	.00	.01	.00	.00
Sphingobium	.01	.01	.17	.80	.08	.33
Sphingomonas	.02	.02	.02	.05	.07	.47
Trichodesmium	.02	.08	.01	.05	.04	.14
Cystobacter	.00	.00	.00	.02	.00	.00
Deferribacter	.00	.00	.00	.00	.00	.00
Desulfotomaculum	.00	.00	.00	.00	.00	.00
Escherichia	.20	.39	.07	.17	.13	.42
Gemella	.01	.01	.02	.10	.07	.47
Legionella	.00	.00	.00	.01	.00	.00
Pediococcus	.00	.00	.00	.00	.00	.00
Planomicrobium	.00	.00	.01	.03	.00	.00
Ralstonia	.00	.00	.01	.03	.01	.03
Rhodococcus	.01	.04	.06	.25	.01	.08
Thermobaculum	.00	.01	.00	.00	.01	.01
Thiomonas	.01	.02	.00	.00	.00	.01
Anaerofustis	.00	.00	.00	.00	.00	.00
Anaerovibrio	.00	.00	.00	.00	.01	.04

Asticcacaulis	.00	.01	.00	.01	.00	.01
Bacillus	.01	.01	.03	.16	.00	.01
Bradyrhizobium	.01	.02	.01	.02	.01	.03
Bulleidia	.01	.01	.01	.02	.02	.08
Citrobacter	.02	.06	.02	.09	.38	2.58
Cohnella	.03	.08	.02	.02	.03	.10
Coriobacterium	.00	.00	.00	.02	.00	.00
Devosia	.00	.00	.01	.08	.00	.00
Granulicatella	.01	.02	.01	.03	.02	.03
Haemophilus	.00	.00	.01	.02	.00	.01
Mitsuokella	.00	.00	.01	.07	.02	.13
Myroides	.00	.00	.03	.19	.00	.00
Niastella	.00	.00	.01	.00	.01	.03
Ochrobactrum	.00	.00	.00	.01	.01	.03
Parascardovia	.02	.02	.02	.02	.02	.03
Rhodobacter	.01	.01	.01	.02	.01	.04
Rikenella	.00	.01	.00	.00	.00	.00
Sarcina	.05	.08	.10	.23	.13	.44
Sphingopyxis	.00	.00	.01	.04	.01	.03
Succiniclasticum	.00	.00	.00	.00	.02	.10
Variovorax	.01	.02	.00	.01	.00	.01
Acidovorax	.09	.35	.14	.82	.01	.03
Acinetobacter	.06	.17	.08	.27	.12	.42
Aerococcus	.00	.00	.17	1.25	.00	.01
Agrobacterium	.00	.00	.01	.04	.07	.36
Akkermansia	.01	.02	.00	.02	.01	.07
Alicycliphilus	.00	.00	.01	.10	.00	.02
Anoxybacillus	.32*	1.30	.04	.27	.00	.00
Burkholderia	.05	.11	.05	.24	.02	.05
Caloramator	.00	.00	.00	.00	.00	.00
Catenibacterium	.25	.80	.29	1.09	.11	.55
Chroococcus	.01	.02	.03	.08	.01	.01
Citricoccus	.00	.00	.01	.05	.00	.00
Cloacibacillus	.01	.06	.01	.05	.00	.00
Comamonas	.03	.13	.17	.73	.40	2.38
Curvibacter	.00	.00	.00	.01	.00	.01
Dechloromonas	.00	.00	.01	.06	.00	.00
Delftia	.04	.11	.23	1.19	.18	.97
Denitrobacter	.72	2.93	.02	.18	.07	.56
Erwinia	.00	.01	.00	.00	.01	.03
Ferrimicrobium	.00	.00	.00	.01	.00	.01
Fusibacter	.00	.00	.01	.02	.01	.05
Gallionella	.01	.02	.01	.02	.00	.01
Giesbergeria	.00	.00	.00	.01	.01	.05
Gluconacetobacter	.00	.00	.00	.01	.03	.21
Haloanella	.00	.00	.07	.55	.00	.00
Herbaspirillum	.00	.01	.00	.01	.01	.01
Hydrogenophaga	.00	.00	.03	.24	.00	.01
Klebsiella	.46*	1.77	.01	.05	.01	.03
Leptothrix	.00	.00	.00	.03	.00	.00
Leucobacter	.01	.02	.00	.01	.00	.01
Leuconostoc	.02	.05	.01	.02	.05	.29
Luteimonas	.00	.00	.01	.04	.00	.01
Macrococcus	.00	.00	.00	.01	.00	.02

Mesorhizobium	.02	.07	.01	.04	.00	.01
Methylibium	.00	.00	.05	.35	.00	.00
Mycoplana	.00	.00	.01	.04	.00	.00
Olivibacter	.00	.00	.00	.02	.01	.05
Phenylobacterium	.00	.00	.11	.78	.00	.00
Plesiomonas	.03	.12	.00	.00	.01	.05
Proteus	.00	.00	.02	.14	.00	.01
Providencia	.00	.01	.00	.01	.00	.01
Pseudochrobactrum	.00	.00	.00	.00	.02	.12
Pseudoclavibacter	.00	.01	.00	.01	.00	.02
Pseudoxanthomonas	.02	.09	.05	.38	.00	.03
Psychrobacter	.00	.00	.22	1.68	.05	.40
Rhizobium	.00	.01	.01	.03	.01	.02
Rhodoferax	.00	.00	.01	.04	.00	.00
Rhodoplanes	.00	.01	.00	.00	.01	.05
Rothia	.00	.00	.00	.01	.00	.01
Sediminibacterium	.00	.00	.00	.02	.00	.00
Serratia	.02	.04	.02	.05	.04	.21
Shinella	.00	.00	.01	.05	.01	.07
Sneathia	.00	.00	.00	.03	.07	.44
Solibacillus	.00	.00	.09	.63	.00	.00
Sphingobacterium	.00	.01	.04	.15	.12	.57
Sporolactobacillus	.00	.00	.01*	.01	.00	.00
Stenotrophomonas	.03	.08	.18	.85	.11	.65
Succinivibrio	.08	.32	.00	.00	.01	.06
Thauera	.00	.00	.03	.24	.00	.00
Thermomonas	.00	.00	.05	.38	.00	.00
Vagococcus	.00	.02	.00	.01	.02	.10
Vibrio	.00	.01	.00	.01	.00	.01
Viridibacillus	.03	.14	.03	.13	.01	.03
Xanthomonas	.02	.09	.04	.30	.01	.03
Zobellia	.00	.01	.00	.01	.00	.00
